# Nanotechnology: Nano–Food Chain Link Examined

**DOI:** 10.1289/ehp.116-a336a

**Published:** 2008-08

**Authors:** Adrian Burton

Engineered nanomaterials—materials and even machines constructed on a nanometer scale—have enormous potential in everything from electronics to textiles to medicine. Although nanomaterials are being manufactured in ever greater numbers, little is known about their biological effects, including whether they biomagnify as they travel up food chains. Now chemical engineer David Holbrook and colleagues at the U.S. National Institute of Standards and Technology report that certain nanomaterials may not accumulate in organisms at higher trophic levels. However, the researchers are quick to add that much more work is required before any generalizations can be made regarding environmental and human safety of nanomaterials.

Holbrook’s team prepared an aqueous environment in the laboratory in which *Escherichia coli* provided a food source for the ciliate *Tetrahymena pyriformis*, which in turn provided a food source for the rotifer *Brachionus calyciflorus*. “This simple food chain represents what we might see in a real aquatic environment,” explains Holbrook. The team then added two types of nanomaterial to their experimental environment: carboxylated and biotinylated quantum dots (QDs) made from cadmium, selenium, zinc and sulfate, whose fluorescing properties make them easy to detect microscopically.

The bacteria did not accumulate the QDs, although the ciliates did. Even so, both types of QDs accumulated at about 21–30% of the benchmark rate at which a pollutant is considered to be “very bioaccumulative.”

The researchers also observed that intact QDs appeared within the gut and body cavity of the rotifers. But whether a contaminant biomagnifies depends in part on how quickly an organism eliminates the contaminant from its body. The researchers determined this rate by placing rotifers that had assimilated QDs into a clean environment, then measuring the fall in their QD content (“depuration”) over time. Holbrook says the depuration rates observed were an order of magnitude higher than the threshold that predicts biomagnification.

However, the study results, which appear in the June 2008 issue of *Nature Nanotechnology*, are not proof that nanomaterials pose no environmental threat. “There are many types of nanomaterials, environments, organisms, and food chains—we have looked at just one type of each,” says Holbrook. “These results are interesting, but extrapolating them very far, such as to natural systems, may not be prudent.”

“More work of this type is essential,” comments Robert Lee, a professor of law at Cardiff University, United Kingdom, and a member of the U.K. Nanotechnology Research Coordination Group. “More attention needs to be paid to the health risks possibly associated with different types of nanomaterial; data on toxicity is essential for governments to properly regulate their use. But information is vital for business, too, in addressing possible legal liability in the future. Companies manufacturing or using nanomaterials in their products need to track this type of work to avoid finding themselves liable for damages should nanoparticles be later shown to cause harm to human health or the environment.”

“These are interesting findings,” agrees Rosa Ortega, a professor of nutrition at Complutense University in Madrid, Spain, “but the properties of different nanomaterials, the different organisms in different food chains, environmental conditions, and how organisms break down different nanomaterials all influence whether they will be bio-magnified. We need to continue work to ensure nanomaterials are safely used.”

## Figures and Tables

**Figure:**
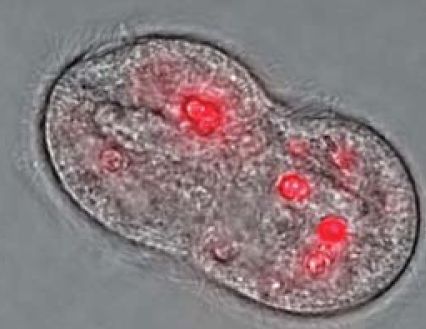
Fluorescing quantum dots in *T. pyriformis*

